# Association of Tobacco Smoking with Physical Fitness of Military Males in Taiwan: The CHIEF Study

**DOI:** 10.1155/2020/5968189

**Published:** 2020-01-07

**Authors:** Fang-Ying Su, Sheng-Huei Wang, Henry Horng-Shing Lu, Gen-Min Lin

**Affiliations:** ^1^Institute of Statistics, National Chiao Tung University, Hsinchu City, Taiwan; ^2^Biotechnology R&D Center, National Taiwan University Hospital Hsinchu Branch, Hsinchu 300, Taiwan; ^3^Division of Pulmonary and Critical Care Medicine, Department of Internal Medicine, Tri-Service General Hospital, National Defense Medical Center, Taipei, Taiwan; ^4^Department of Internal Medicine, Hualien Armed Forces General Hospital, Hualien County, Taiwan; ^5^Department of Internal Medicine, Tri-Service General Hospital, National Defense Medical Center, Taipei, Taiwan; ^6^Department of Preventive Medicine, Northwestern University Feinberg School of Medicine, Chicago, IL 60611, USA

## Abstract

Tobacco smoking has been found associated with lower cardiorespiratory fitness in white and black males; however, few studies have not been conducted to clarify such relationship in Asian males. We performed a cross-sectional study to investigate the association between tobacco smoking status and physical fitness in 3,669 military males, averaged 29.4 years of age, from the cardiorespiratory fitness and hospitalization events in armed forces (CHIEF) study in Taiwan during 2014. There were 1,376 current smokers, and the others were noncurrent smokers. The effective sample size estimated was 1,230 participants, as the margin of error was ±3% at the 99% confidence level. Physical fitness was evaluated by time for a 3000-meter run test (aerobic fitness) and repetitive numbers of 2-minute sit-ups and 2-minute push-ups (anaerobic fitness) where all procedures were standardized by using computerized scoring systems. A multiple linear analysis adjusting for age, service specialty, body mass index, heart rate, alcohol intake, and training frequency was used to determine the relationship. As compared with noncurrent smoking, current smoking was inversely correlated with longer time for a 3000-meter run (*β* = 15.66 (95% confidence intervals (CI): 10.62, 20.70)) and fewer repetitive numbers of 2-minute sit-ups and 2-minute push-ups (*β* = −1.53 (95% CI: −2.08, −0.97) and −1.31 (95% CI: −2.12, −0.50), respectively). Our finding reconfirms the concept that tobacco smoking might reduce both aerobic and anaerobic fitness among young Asian males.

## 1. Introduction

The global prevalence of daily tobacco smoking in 2015 is 25.0% and 5.4% for men and women, respectively [1]. To our best knowledge, the prevalence (20%–66%) of tobacco use in military is a longstanding problem worldwide [2, 3]. In Taiwan, although the prevalence of tobacco smoking among military personnel has been decreasing in the past decades after application of various smoking cessation programs [4], the prevalence of active smoking in military remained over 30% in 2014. Smoking is associated with cancers, cardiovascular diseases, and respiratory diseases and is the second leading cause for disability and premature death in the world in 2015 [5]. In addition, secondhand tobacco smoke exposure also causes coronary heart disease and the related morality in nonsmokers [6], which as a whole results in enormous societal cost and public health burden, estimated 422 billion US dollars on global health care expenses attributable to smoking in 2012 [7].

With regard to cardiorespiratory function, De and Tripathi investigated the effect of smoking on the lung function of sportsmen, showing that current smokers had lower expiratory airflow with a 7–12% reduction of FEV1 (forced expiratory volume in the first second), the ratio of FEV1 to FVC (forced vital capacity), and peak expiratory flow rate compared with nonsmokers [8]. In addition, Eroglu et al. used Doppler myocardial imaging to reveal mild impairment of systolic and diastolic functions of right and left ventricles in healthy young smokers [9]. Furthermore, de Borba et al. reported that nonsmokers had higher maximum oxygen consumption relative to active smokers and passive smokers in asymptomatic adults during a submaximal exertion incremental test on a treadmill, indicating that tobacco smoking may reduce cardiorespiratory fitness [10]. Experimental studies of mice also demonstrated that exposures of smoke might alter specific intestinal microbial growths [11] and result in cardiac contractile function impairment that could be attenuated by some cytokines such as adiponectin through facilitating autophagolysosome formation [12].

Previous studies have reported that Asian males might have less smoking-related annual excess FEV1 declines, estimated 3 mL per pack years, as compared with other racial males [13, 14]. In a Japanese male cohort, smoking was not associated with maximal oxygen uptake during treadmill exercise as well [15]. It is reasonable that the declines in lung function could lead to poor exercise performance. Although the evidence for an adverse impact of smoking on physical fitness has been confirmed in white and black populations, the relationship remained unclear in Asian males. Therefore, we investigated the association of smoking with aerobic and anaerobic fitness in a large military male cohort in Taiwan to clarify the adverse effect of smoking on fitness in Asian males.

## 2. Methods

### 2.1. Study Population

**Figure 1:**
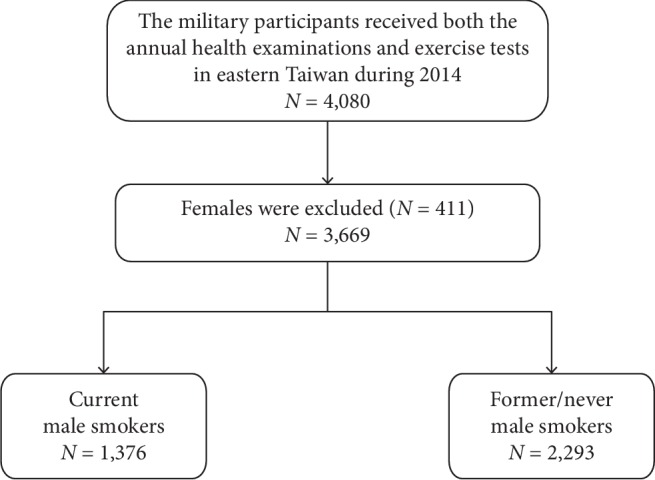


The retrospective cohort was obtained from the Cardiorespiratory Fitness and Hospitalization Events in Armed Forces (CHIEF) study during 2014 [16–21]. The design of the study has been described in detail previously [16]. In summary, the present study included overall 4,080 military personnel in Eastern Taiwan, aged 18–50 years, received the annual health examination in the Hualien-Armed Forces General Hospital, and participated in at least one of the three exercise tests for their military rank promotions or awards at the Military Physical Training and Testing Center in 2014. All females (*n* = 411) were excluded for not being the subjects of interest, leaving a sample of 3,669 males for the analysis, and the flowchart showing the study subjects selection is shown in Figure 1.

### 2.2. Measurements

Measurements of height and weight of each participant were performed in a standing position. Body mass index was calculated as body weight (kg) divided by height squared (m^2^), and waist circumference was assessed at the midpoint between the lowest palpable rib and the top of the iliac crest. Blood pressure and pulse rate of each participant were measured once in a sitting position over the right upper arm by using an automated blood pressure monitor (FT-201, Parama-Tech Co. Ltd., Fukuoka, Japan) after a break for 15 minutes or longer. Systemic arterial hypertension was defined as systolic blood pressure ≥140 mmHg, and/or, diastolic blood pressure ≥90 mmHg, or on antihypertensive therapy according to the guideline of JNC VII [22].

### 2.3. Smoking Status Assessment

During the annual health examination, all participants were interviewed with a military physician and self-reported a questionnaire for their tobacco smoking status (current vs. former or never) in the past 10 years. In addition, the status of alcohol consumption (current vs. former or never) and exercise frequency (endurance or resistant exercises performed longer than 30 minutes, times/per week) in leisure times in the past 6 months were also evaluated in the questionnaire.

### 2.4. Physical Fitness Tests

Anaerobic fitness was separately evaluated by the 2-minute push-up and 2-minute sit-up tests where the stopping point (2 minutes) in brief bursts of exercises was determined by the findings of previous studies [23, 24]. The two procedures were performed on sponge pads and standardized by using computerized scoring systems. The push-up movement was scored when the examinee's back returned to the initial resting set level in a line with head and buttocks, simultaneously detected by using infrared sensors within 2 minutes. However, the push-up test was stopped immediately upon the body going down on the pad before the time ran out. With regard to the 2-minute sit-up test, the examinees were prepared with their feet fixed by the anchor on the floor and both hands close to the ears. It was scored only when their upper trunk bended forward and elbows touched the artificial sensors on both thighs. If the examinees' hands left the ears temporarily, the test would be cancelled immediately by the supervisors. Aerobic fitness was evaluated by the 3000-meter run test. The examinees ran on a flat playground at the Military Physical Training and Testing Center and did not carry any heavy object. The test was performed outdoor at 16:00 PM uniformly if the coefficient obtained from the heat stroke risk formula was <40 (the product of outdoor temperature (°C) and relative humidity (%) × 0.1) and without heavy raining. This study was reviewed and approved by the Institutional Review Board of the Mennonite Christian Hospital (16-05-008) in Taiwan, and written informed consent was obtained from all participants.

### 2.5. Statistical Analysis

Continuous variables were presented as mean ± standard deviation (SD) and compared by the two-tailed *t*-test when the Kolmogorov–Smirnov test was fulfilled; otherwise, the Wilcoxon signed-rank test was used. Categorical variables were expressed as numbers (percentages) and compared by *χ*^2^ test or Fisher's exact test. The difference in each exercise performance between current and noncurrent smokers was estimated by using analysis of covariance (ANCOVA), and the results were expressed as mean ± standard error (SE). Multiple linear regression analyses were used to determine the relationship of current smoking with aerobic and anaerobic fitness. In addition, multiple logistic regressions were used to determine the odds ratio (OR) of the best 10% performers and the worst% performers in each exercise with current smoking status to noncurrent smoking status. In model 1, age, service specialty, pulse rate, and alcohol intake status were adjusted. In model 2, hypertension status and exercise frequency were additionally adjusted. A 2-tailed value of *p* < 0.05 was considered significant. SAS statistical software (SAS System for Windows, version 9.4; SAS Institute, Cary, NC, United States) was used for all statistical analyses.

## 3. Results

### 3.1. Baseline Group Characteristics

Table 1 shows the baseline characteristics of the participants. There were 1,376 participants who were current smokers (37.5%), and the other 2,293 participants did not have current smoking. The effective sample size estimated was 1,230 participants as the margin of error was ±3% at the 99% confidence level. The current and noncurrent smokers had similar age, body mass index, and exercise frequency at baseline. However, the current smokers had faster pulse rate and lower diastolic blood pressure and higher prevalence of alcohol intake than the noncurrent smokers.

### 3.2. Group Mean Comparisons

Table 2 reveals that there were significant differences in repetitive numbers of 2-minute push-ups and 2-minute sit-ups performed and time for a 3000-meter run between current smokers and noncurrent smokers after stepwise adjusting for the potential covariates in models 1 and 2. In general, current smokers had superior aerobic and anaerobic fitness than nonsmokers.

### 3.3. Multiple Linear Regression

The multiple linear regression results of each exercise performance, with current smoking relative to noncurrent smoking, in models 1 and 2 are shown in Table 3. The relationships between current smoking and each exercise performance are in line with the findings presented in Table 2. In model 1, current tobacco smoking was significantly correlated with fewer numbers of 2-minute push-ups and 2-minute sit-ups performed (*β* = −1.25 and −1.53, respectively; both *p* < 0.01) and correlated with longer time for a 3000-meter run (*β* = 14.03; *p* < 0.0001). In model 2, the associations of current tobacco smoking with numbers of 2- minute push-ups and 2-minute sit-ups performed and time for a 3000-meter run remained significant (*β* = −1.31, −1.53, and 15.66, respectively; all *p* < 0.01).

### 3.4. Multiple Logistic Regression

Table 4 reveals the multiple logistic regression results for the relationship of smoking with the best 10% and the worst 10% performers in each exercise, respectively. After the adjustments in model 1 and model 2, the current smokers had lower possibility as the best 10% performers in the tests of 2-minute push-ups (OR: 0.59 and 0.58, respectively; both *p* < 0.0001) and 2-minute sit-ups (OR: 0.58 and 0.58, respectively; both *p* < 0.0001) as compared with the noncurrent smokers. On the other hand, the current smokers had higher possibility to be the worst 10% performers in the tests of 3000-meter run (OR: 1.39 and 1.42, respectively; both *p* < 0.01) and 2-minute sit-ups (OR: 1.39 and 1.43, respectively; both *p* < 0.01) when compared with the noncurrent smokers.

## 4. Discussion

Our principal finding was that active tobacco smoking was associated with lower aerobic and anaerobic fitness in a large military Asian cohort of young and middle aged males. Since this was the first study investigating the relationship between the tobacco smoking status and the exercise performances among Asian males, the finding filled the gap for the concept that smoking could reduce physical fitness despite a possible interaction of sex and race/ethnicity on this relationship.

Several studies conducted in the Western countries have uncovered the adverse impacts of active smoking on physical fitness, which were evaluated by the posttraining performance or the differences between posttraining and pretraining performance in strength and endurance exercises in the military. An UK study reported that in 165 British officer cadets receiving a 6-month physical training program, both the smokers and nonsmokers improved their fitness but the nonsmokers were less fit when compared with the smokers [25]. Another UK study carried out by Siddall et al. also reported that in 1,182 military subjects, the current smokers had lower muscular (fewer numbers of 2 minute push-ups and sit-ups) and cardiorespiratory endurance performances (longer time for a 2.4 km run) than the nonsmokers at baseline and after a 24-week training program; however, the improvements in both aerobic and anaerobic fitness tended to be larger in the smokers than the nonsmokers [26]. In a Swiss study, among 6,592 army conscripts, daily tobacco consumption and years of tobacco smoking were inversely associated with the distance covered in a 12 min endurance run [27]. In addition, a US study demonstrated that in 3,045 navy personnel, smoking was inversely associated with cardiorespiratory (1.5 mile run) and muscular (sit-ups) endurance after controlling for exercise activity [28].

The mechanisms for the risk of low physical fitness in smokers have been hypothesized. First, habitual smoking could cause lower gas change efficacy in the muscle and lung. Kobayashi et al. have shown that blood lactate concentrations increased higher in chronic smokers at maximal workload of exercise, indicating a reduction of oxygen extraction because of the carbon monoxide effect [29]. Second, active smoking can damage the vascular endothelium and induce free radicals that influence the respiratory muscle blood supply, reduce the strength of respiratory muscle, and subsequently result in poor lung function [30, 31]. Third, habitual smoking induces inflammation and increases oxidative stress, contributing to vascular thrombosis and cardiovascular dysfunction [32]. Fourth, tobacco smoke and the associated systemic inflammatory mediators promote proteolysis, suppress protein synthesis, and impair oxygen delivery to the mitochondria, which attenuate the ability of mitochondria to generate ATP and lead to reduced muscle mass and skeletal muscle contractile endurance [33].

There were some strengths in this study. First, there were large numbers of male subjects to provide a sufficient power to detect the difference in the relationship between smoking status and physical fitness. Second, all exercise tests were performed in a strict manner and the processes were standardized. Third, several important covariates have been controlled in the multivariable models to avoid the bias. In addition, many unmeasured confounding factors in military had been strictly controlled at baseline since the daily life in military was unified. On the contrary, there remained some limitations in our study. First, although the tobacco smoking status was classified to be current versus former or never, we could not further analyze the impact of daily dose and the amount of time of smoking on physical fitness. Second, we did not perform lung function test for the participants at baseline, which was a vital mediator for the relationship between current smoking and physical fitness. Third, response bias in self-report measures for the tobacco smoking status might exist because of personal considerations to being good in military. Fourth, we did not have the initial level of physical fitness in each participant before military training at enlist and could not exclude the possibility that current smokers might have a better fitness at baseline. Fifth, there were more prevalence of active alcohol intake among current smokers, and heavy amount of alcohol beverages intake might reduce endurance exercise performance, cardiorespiratory fitness, and cause muscle fatigue [34, 35], possibly overestimating the adverse impact of tobacco smoking on physical fitness, despite an adjustment for the status of alcohol consumption.

In conclusion, our finding filled the gap of the concept that active smoking might reduce both aerobic and anaerobic fitness among young and middle-aged Asian males as well. Therefore, it is important to implement the policy of smoking cessation strictly in military in order to building fit and healthy armed forces.

## Figures and Tables

**Table 1:** Baseline characteristics and exercise performances of noncurrent smokers and current smokers.

Characteristics	Overall	Noncurrent smokers	Current smokers	*p* value^†^
	*N* = 3,669	*N* = 2,293	*N* = 1,376	
Age (years)	29.35 ± 5.88	29.36 ± 5.88	29.33 ± 5.88	0.88
Service specialty (%)				<0.0001
Air forces	1,027 (27.99)	633 (27.61)	394 (28.63)	
Army	1,854 (50.53)	1.216 (53.03)	638 (46.37)	
Navy	788 (21.48)	444 (19.36)	344 (25.00)	
Heart rate (times/min)	72.15 ± 10.81	71.31 ± 10.63	73.55 ± 10.95	<0.0001
BMI (kg/m^2^)	24.87 ± 3.10	24.89 ± 3.03	24.83 ± 3.21	0.56
Waist circumference (cm)	83.39 ± 7.95	83.36 ± 7.80	83.45 ± 8.20	0.74
Systolic BP (mmHg)	118.38 ± 13.10	118.65 ± 13.07	117.94 ± 13.14	0.11
Diastolic BP (mmHg)	70.58 ± 10.12	71.02 ± 10.07	69.84 ± 10.16	0.0006
Hypertension (%)	322 (8.78)	200 (8.72)	122 (8.87)	0.88
Current alcohol intake (%)	1,616 (44.04)	710 (30.96)	906 (65.84)	<0.0001
Frequency of exercise (%)				0.23
Never or occasionally	769 (20.96)	473 (20.63)	296 (21.51)	
1-2 (times/week)	1,378 (37.56)	844 (36.81)	534 (38.81)	
≥3 (times/week)	1,522 (41.48)	976 (42.56)	546 (39.68)	

^†^Data were presented as the mean ± SD for continuous data and percentage (%) for categorical data, which were compared by the *t*-test and chi-square test, respectively. BMI, body mass index; BP, blood pressure. Body mass index was defined as weight (kg)/height squared (m^2^).

**Table 2:** Difference in each exercise performance between current smoker and noncurrent smoker.

	2 min push-ups (numbers)			2 min sit-ups (numbers)			3000 m running (seconds)		
	*N*	Mean ± SE	*p* value	*N*	Mean ± SE	*p* value	*N*	Mean ± SE	*p* value
Unadjusted									
Current smoker	1,366	48.21 ± 0.30	<0.0001	1,365	46.51 ± 0.15	0.0006	1,183	868.79 ± 2.09	<0.0001
Noncurrent smoker	2,275	49.55 ± 0.25		2,286	48.08 ± 0.23		2,113	854.94 ± 1.58	
Model 1									
Current smoker	1,361	48.46 ± 0.26	0.0031	1,360	46.59 ± 0.22	<0.0001	1,178	868.05 ± 2.09	<0.0001
Noncurrent smoker	2,261	49.72 ± 0.32		2,273	48.12 ± 0.18		2,102	854.04 ± 1.68	
Model 2									
Current smoker	1,361	48.27 ± 0.43	0.0014	1,360	46.28 ± 0.29	<0.0001	1,178	872.22 ± 2.74	<0.0001
Noncurrent smoker	2,260	49.59 ± 0.39		2,272	47.82 ± 0.26		2,101	856.59 ± 2.43	

Mean (standard errors, SE) for each exercise performance estimated using analysis of covariance with adjustments for model 1 adjusted for age, heart rate, specialty, and alcohol drinking and model 2 adjusted for age, body mass index, heart rate, specialty, hypertension, alcohol drinking, and exercise frequency.

**Table 3:** Multiple liner regressions of current smoking with each exercise performance.

	*Β*	SE	(95% CI)		*p* value
Model 1					
2 min push-ups	−1.25	0.42	−2.08	−0.42	0.0033
2 min sit-ups	−1.53	0.29	−2.09	−0.97	<0.0001
3000 m running	14.03	2.70	8.73	19.32	<0.0001
Model 2					
2 min push-ups	−1.31	0.41	−2.12	−0.50	0.0016
2 min sit-ups	−1.53	0.28	−2.08	−0.97	<0.0001
3000 m running	15.66	2.57	10.62	20.70	<0.0001

Data are presented as *β* (SE, standard error and 95% CI, confidence interval) for model 1 adjusted for age, heart rate, specialty, and alcohol drinking and model 2 adjusted for age, body mass index, heart rate, specialty, hypertension, alcohol drinking, and exercise frequency.

**Table 4:** Logistic regression models comparing current smokers with noncurrent smokers by the best 10% and the worst 10% exercise performances, respectively.

	OR	95% CI	*p* value
*Top 10% performance levels*			
Model 1			
2 min push-up ≥60 times	0.590	0.464 to 0.751	<0.0001
2 min sit-up ≥58 times	0.582	0.450 to 0.752	<0.0001
3000 m run ≤783 sec	1.024	0.853 to 1.229	0.80
Model 2			
2 min push-up ≥60 times	0.578	0.454 to 0.737	<0.0001
2 min sit-up ≥58 times	0.581	0.449 to 0.752	<0.0001
3000 m run ≤783 sec	1.030	0.857 to 1.237	0.76

*Bottom 10% performance levels*			
Model 1			
2 min push-up ≤37 times	1.107	0.878 to 0.878	0.39
2 min sit-up ≤40 times	1.392	1.093 to 1.774	0.0074
3000 m run ≥934 sec	1.386	1.084 to 1.772	0.0092
Model 2			
2 min push-up ≤37 times	1.122	0.885 to 1.423	0.34
2 min sit-up ≤40 times	1.420	1.110 to 1.817	0.0053
3000 m run ≥934 sec	1.428	1.110 to 1.837	0.0056

OR, odds ratio; CI, confidence intervals. Multiple logistic regression analysis was used to determine the smoking association with the best 10% and the worst 10% exercise performance. Model 1 was adjusted for age, heart rate, specialty, and alcohol drinking. Model 2 was adjusted for age, body mass index, heart rate, service specialty, hypertension, alcohol drinking, and exercise frequency.

## Data Availability

The data were obtained from a military cohort in Taiwan. If there is any problem with the results of the paper and the raw data need to be reanalyzed, please contact the corresponding author. Since this is a military population study, there are some secure data unavailable in public.

## Abbreviations

CHIEF study: Cardiorespiratory Fitness and Hospitalization Events in Armed Forces study
FEV1: Forced expiratory volume in the first second
FVC: Forced vital capacity.

## References

[1] Reitsma M. B., Fullman N., Ng M. (2017). Smoking prevalence and attributable disease burden in 195 countries and territories, 1990–2015: a systematic analysis from the global burden of disease study 2015. *The Lancet*.

[2] Al-Khashan H. I., Al Sabaan F. S., Al Nasser H. S. (2014). The prevalence of smoking and its associated factors among military personnel in Kingdom of Saudi Arabia: a national study. *Journal of Family & Community Medicine*.

[3] Hussain N., Akande T., Adebayo O. (2010). Prevalence of cigarette smoking and knowledge implications among Nigerian soldiers of its health. *East African Journal of Public Health*.

[4] Chu N.-F., Lin F.-H., Wu Y.-C. (2017). Prevalence and trends of cigarette smoking among military personnel in Taiwan: results of 10-year anti-smoking health promotion programs in military. *Military Medicine*.

[5] Forouzanfar M. H., Afshin A., Alexander L. T. (2016). Global, regional, and national comparative risk assessment of 79 behavioural, environmental and occupational, and metabolic risks or clusters of risks, 1990–2015: a systematic analysis for the global burden of disease study 2015. *The Lancet*.

[6] Kawachi I., Colditz G. A., Speizer F. E. (1997). A prospective study of passive smoking and coronary heart disease. *Circulation*.

[7] Goodchild M., Nargis N., Tursan d’Espaignet E. (2018). Global economic cost of smoking-attributable diseases. *Tobacco Control*.

[8] De A. K., Tripathi M. M. (1988). Smoking and lung functions in sportsmen. *British Journal of Sports Medicine*.

[9] Eroglu E., Aydin S., Yalniz F., Kalkan A. K., Bayrak F., Degertekin M. (2009). Chronic cigarette smoking affects left and right ventricular long-axis function in healthy young subjects: a doppler myocardial imaging study. *Echocardiography*.

[10] de Borba A., Jost R., Gass R. (2014). The influence of active and passive smoking on the cardiorespiratory fitness of adults. *Multidisciplinary Respiratory Medicine*.

[11] Wang H., Zhao J. X., Hu N., Ren J., Du M., Zhu M. J. (2012). Side-stream smoking reduces intestinal inflammation and increases expression of tight junction proteins. *World Journal of Gastroenterology*.

[12] Hu N., Yang L., Dong M., Ren J., Zhang Y. (2015). Deficiency in adiponectin exaggerates cigarette smoking exposure-induced cardiac contractile dysfunction: role of autophagy. *Pharmacological Research*.

[13] Vollmer W. M., Enright P. L., Pedula K. L. (2000). Race and gender differences in the effects of smoking on lung function. *Chest*.

[14] Dransfield M. T., Davis J. J., Gerald L. B., Bailey W. C. (2006). Racial and gender differences in susceptibility to tobacco smoke among patients with chronic obstructive pulmonary disease. *Respiratory Medicine*.

[15] Kawabata K., Imaki M., Ohguri M., Kondo H., Hayashi Y., Tanada S. (1997). Study on the relationship between lifestyles and maximal oxygen uptake in healthy adults. *Japanese Journal of Hygiene*.

[16] Lin G.-M., Li Y.-H., Lee C.-J. (2016). Rationale and design of the cardiorespiratory fitness and hospitalization events in armed forces study in eastern Taiwan. *World Journal of Cardiology*.

[17] Chen Y.-J., Chen K.-W., Shih Y.-L. (2017). Chronic hepatitis B, nonalcoholic steatohepatitis and physical fitness of military males: CHIEF study. *World Journal of Gastroenterology*.

[18] Chen K. W., Meng F. C., Shih Y. L. (2018). Sex-specific association between metabolic abnormalities and elevated alanine aminotransferase levels in a military cohort: the CHIEF study. *International Journal of Environmental Research and Public Health*.

[19] Chao W. H., Su F. Y., Lin F., Yu Y. S., Lin G. M. (2019). Association of electrocardiographic left and right ventricular hypertrophy with physical fitness of military males: the CHIEF study. *European Journal of Sport Science*.

[20] Tsai K. Z., Lai S. W., Hsieh C. J. (2019). Association between mild anemia and physical fitness in a military male cohort: the CHIEF study. *Scientific Reports*.

[21] Lu S. C., Liu F. Y., Hsieh C. J. (2019). Quantitative physical fitness measures inversely associated with myopia severity in military males: The CHIEF Study. *American Journal of Mens Health*.

[22] Medbø J. I., Mohn A. C., Tabata I., Bahr R., Vaage O., Sejersted O. M. (1988). Anaerobic capacity determined by maximal accumulated O_2_ deficit. *Journal of Applied Physiology (1985)*.

[23] Spurway N. C. (1992). Aerobic exercise, anaerobic exercise and the lactate threshold. *British Medical Bulletin*.

[24] Hoad N. A., Clay D. N. (1992). Smoking impairs the response to a physical training regime: a study of officer cadets. *Journal of the Royal Army Medical Corps*.

[25] Siddall A. G., Bilzon J. L. J., Thompson D., Greeves J., Izard R., Stokes K. A. (2017). Smoking status and physical fitness during initial military training. *Occupational Medicine*.

[26] Marti B., Theodor A., Minder C. E., Vader J. P. (1988). Smoking, alcohol consumption, and endurance capacity: an analysis of 6,500 19-year-old conscripts and 4,100 joggers. *Preventive Medicine*.

[27] Conway T. L., Cronan T. A. (1992). Smoking, exercise, and physical fitness. *Preventive Medicine*.

[28] Kobayashi Y., Takeuchi T., Hosoi T., Loeppky J. A. (2004). Effects of habitual smoking on cardiorespiratory responses to sub-maximal exercise. *Journal of Physiological Anthropology and Applied Human Science*.

[29] Holmen T. L., Barrett-Connor E., Clausen J., Holmen J., Bjermer L. (2002). Physical exercise, sports, and lung function in smoking versus nonsmoking adolescents. *European Respiratory Journal*.

[30] Tantisuwat A., Thaveeratitham P. (2014). Effects of smoking on chest expansion, lung function, and respiratory muscle strength of youths. *Journal of Physical Therapy Science*.

[31] Ambrose J. A., Barua R. S. (2004). The pathophysiology of cigarette smoking and cardiovascular disease: an update. *Journal of the American College of Cardiology*.

[32] Degens H., Gayan-Ramirez G., van Hees H. W. H. (2015). Smoking-induced skeletal muscle dysfunction: from evidence to mechanisms. *American Journal of Respiratory and Critical Care Medicine*.

[33] Vella L. D., Cameron-Smith D. (2010). Alcohol, athletic performance and recovery. *Nutrients*.

[34] Barnes M. J., Mündel T., Stannard S. R. (2010). Acute alcohol consumption aggravates the decline in muscle performance following strenuous eccentric exercise. *Journal of Science and Medicine in Sport*.

[35] Bray I., Richardson P., Harrison K. (2013). Smoking prevalence amongst UK armed forces recruits: changes in behaviour after 3 years follow-up and factors affecting smoking behaviour. *Journal of the Royal Army Medical Corps*.

